# Diversity of lactic acid bacterial in *inasua* fermentation

**Published:** 2018-10

**Authors:** Ferymon Mahulette, Nisa Rachmania Mubarik, Antonius Suwanto, Widanarni

**Affiliations:** 1 Study Program of Microbiology, Graduate School of Bogor Agricultural University, Bogor, Indonesia; 2 Department of Biology, Faculty of Mathematics and Natural Sciences, Bogor Agricultural University, Bogor, Indonesia; 3 Department of Aquaculture, Faculty of Fisheries and Marine Sciences, Bogor Agricultural University, Bogor, Indonesia

**Keywords:** Denaturing gradient gel electrophoresis, Dominance index, Fermented fish, Succession

## Abstract

**Background and Objectives:**

*Inasua* is one of the traditional fermented fish products in Maluku, Indonesia. There are two types of *inasua*, i.e. with and without sap. The research aimed to study the succession of lactic acid bacteria (LAB) during fermentation and microbial composition in *inasua*.

**Materials and Methods:**

The sample of *inasua* was taken from two traditional producers in Layeni village, Ceram Island. The diversity of lactic acid bacteria was analyzed based on the 16S rRNA gene sequence.

**Results:**

The succession of lactic acid bacteria was strongly influenced by the physicochemical characteristics during fermentation. *Lactobacillus plantarum* was found dominant in both *inasuas* fermentation processes. At end of fermentation, *L. plantarum* was still found dominant in *inasua* with sap while *inasua* without sap was dominated by *Leuconostoc mesenteroides*. In addition, *Lactobacillus paracasei* (LAB) was found only in *inasua* with sap. The result of Denaturing Gradient Gel Electrophoresis (DGGE) revealed that *Lactobacillus* was the dominant bacteria in *inasua* with sap while *Staphylococcus* was dominant in *inasua* without sap.

**Conclusion:**

*Inasua* with sap was found with higher bacterial diversity index and lower evenness and dominance indices, as well as more complex LAB succession pattern during fermentation and bacterial composition, as opposed to *inasua* without sap.

## INTRODUCTION

Fish is the main source of protein consumed by people in Maluku. Fish is often caught abundantly, making preservation necessary. One of the fish preservation techniques with fermentation is *inasua*. It is a local wisdom of Teon, Nila, and Serua communities. In addition to being used as a food reserve during lean times (1) in the past, the fermented product was also used by communities as a food stock during shipping when they sold cloves to other islands. Raw materials used to produce *inasua* are reef fish, salt and coconut sap. However, in certain conditions, producing *inasua* does not always use coconut sap. While *inasua* with coconut sap (*inasua*-S) uses both coconut sap and salt as preservatives, *inasua* without coconut sap (*inasua*-NS) uses only salt. Both have distinct sensory characteristics and different shelf life.

Sensory characteristics and shelf life of a fermented product are strongly influenced by microbial diversity. *Inasua* fermentation occurs spontaneously and involves various types of microorganisms. The main microbe involved in fish fermentation is LAB (2). The bacteria belong to the generally recognized as safe (GRAS) category, making it safe to be in a food product (3). The composition of LAB in traditional fish fermentation is highly determined by the type of carbohydrate and the amount of salt added (4). LAB found in *inasua* fermentation can be developed as a starter in various fermented products.

DGGE is one of the methods for detecting microbial diversity in a fermented product (5). This method can determine the safety aspects of *inasua*. The objective of this research was to study the succession of LAB during fermentation and microbial composition in *inasua*-S and *inasua*-NS.

## MATERIALS AND METHODS

### *Inasua* sampling.

The sample of *inasua* was taken from two traditional producers in Layeni village, TNS Waipia Sub-District, Ceram Island (each produced both *inasua*-S and *inasua*-NS). A total of 5 kg brown stripe red snapper (*Lutjanus vitta* L) was obtained from the sea around the Ceram Island and 2.5 kg was processed into *inasua*-NS by adding salt only and the remaining 2.5 kg into *inasua*-S by adding both salt and coconut sap. The *inasua* was then allowed to ferment in jars at room temperature for 12 weeks. Analysis of LAB succession was carried out in week one until week 12 of fermentation. A 500 g sample from each *inasua* type was taken into the laboratory for further analysis.

### Isolation and characterization of lactic acid bacteria.

A 25 g sample from each *inasua* type was mixed with 225 ml sterile peptone solution and homogenized using stomacher bags. One ml of the homogenized and diluted sample was poured into Petri dishes followed with de Man, Rogosa and Sharp agar (MRSA) media containing 1% CaCO_3_ with 3% and 5% and NaCl prior to incubation at room temperature for 48 hours. All isolates obtained were stained with Gram and spore staining, catalase test, and fermentation of carbohydrates (6).

### Extraction and amplification of LAB’s 16S rRNA.

DNA extraction was carried out following procedure from Presto TM Mini GDNA Kit (Gene-aid). The result of DNA extraction was used to amplify 16S rRNA gene. The 16S rRNA gene was amplified using PCR machine with 63F (5′-CAG GCC TAA CAC ATG CAA GTC-3′) and 1387R (5′-GGG CGG WGT GTA CAA GGC-3′) primers (7). The volume of PCR reaction used was 25 μL, consisting of 12.5 μL GoTaq Green Master Mix 2X (Promega, Madison, W1, USA); 2.5 μL 63F and 1387R primers each (10 pmol); 6.5 μL Nuclease Free Water and 1 μg DNA genome as template. The reaction was amplified in 30 cycles and each PCR comprised pre-denaturation at 95°C for 5 mins, annealing at 55°C for 1 min, elongation at 72°C for 1.5 min, and extension at 72°C for 10 mins. PCR products were visualized using an electrophoresis machine at 80 volts for 45 mins and stained with ethidium bromide.

### Construction of phylogeny tree.

The amplified DNA was further sequenced and analyzed using ChromasPro software (Technelysium, AU) for sequence coupling. The sequences were then compared with GenBank database using Basic Local Alignment Search Total Nucleotide (BLASTN) software. The obtained homologous sequences were then aligned using MEGA 6.0 software (8) with 1000x bootstrap replications while phylogenetic tree was constructed using Neighbor Joining method.

### DNA extraction and amplification of bacterial genomes from *inasua*.

The DNA isolation of all samples that had been fermented for 3 months followed the protocol from Food DNA Isolation Kit (Norgen, Thorold, ON, Canada). Amplification of the 16S rRNA gene used PCR machine to detect bacteria in *inasua* and was performed using P338F-GC (5′-CG-CCCGCCGCGCGCGG-CGGGCGGGG-CGGGGGCCCGGGGGGACTCCGGGAGGCAGCAG-′3) and P518R (5′-ATTA-CCGC-GGCTGCTGG-′3) primers (9). PCR reaction used a mixture consisting of 1 ml DNA template, 2 μl each primer (20 pmol), 1 μl dNTP (100 mM for each dNTP), 5 μl 10× PCR buffers, 0.25 μl *Taq* polymerase and 40.7 μl H_2_O. The reaction was amplified in 30 cycles and each PCR comprised initial denaturation at 94°C for 5 mins, denaturation at 94°C for 30 secs, annealing at 55°C for 30 secs, elongation at 72°C for 30 secs, and extension at 72°C for 7 mins. PCR products were visualized using an electrophoresis machine at 80 volts for 45 mins and stained with ethidium bromide.

### Denaturing Gradient Gel Electrophoresis (DGGE).

Totally, 20 μg DNA from the amplification was mixed with 4 μl loading dye prior to migration through 6% (b/v) polyacrylamide gel in TAE 1× buffer (pH 7, 10 mM sodium acetate, 0.5 mM Na, -EDTA) with a gel prepared from 30–70% (b/v) acrylamide stock solution (acrylamide-N, N’-methylene bisacrylamide, 37.5: 1) containing denatures (100% denatures: 7 M urea and 40% (v/v) formamide) (10).

### Analysis of microbial diversity.

Diversity index was analyzed based on the interpretation of CLIQS ID software while relative abundance and Operational Taxonomic Unit (OTU) dominance values of DGGE results using PAST3 software (11). In addition, microbial diversity was analyzed using Shannon-Wiener diversity index obtained based on the OTU richness and the proportion of abundance of each OTU. The formula of Shannon-Wiener index is H′= −∑ (pi log pi). H = diversity index, pi = the proportion of the number of individuals of an OTU to the total number of individual samples in the plot (n/N) (12).

## RESULTS

### Diversity of culturable lactic acid bacteria.

A total of 50 isolates of lactic acid bacteria were obtained during *inasua* fermentation, comprising 22 isolates from *inasua*-NS and 28 isolates from *inasua*-S. Analysis of gene sequences encoding 16S rRNA from 18 selected isolates (*inasua* -NS: 6; *inasua*-S: 12) with GenBank data using the BLAST-N program revealed that all isolates were LAB that are closely related to the *Lactobacillus* and *Leuconostoc* groups. The percentage of sequence similarity with target 16S rRNA gene in genBank database was 96–99% (Table 1).

**Table 1.** Bacterial isolates obtained from two types of *inasua* fermentation

**Sample**	**Isolate**	**Description**	**Length of nucleotide (bp)**	**Identity**	**Accession**
*Inasua*-NS	ITN-03	*Lactobacillus rhamnosus* NBRC 3425	1320	99%	NR 113332.1
	ITN-05	*Lactobacillus rhamnosus* NBRC 3425	1287	99%	NR 113332.1
	ITN-06	*Lactobacillus plantarum* CIP 103151	1262	99%	NR 104573.1
	ITN-12	*Lactobacillus plantarum* CIP 103151	1277	99%	NR 104573.1
	ITN-13	*Lactobacillus rhamnosus* NBRC 3425	1307	99%	NR 113332.1
	ITN-17	*Leuconostoc mesenteroides* ATCC 8293	1345	99%	NR 074957.1
	IN-01	*Lactobacillus rhamnosus* NBRC 3425	1351	99%	NR 113332.1
*Inasua*-S	IN-02	*Lactobacillus plantarum* JCM 1149	1355	99%	NR 115605.1
	IN-04	*Lactobacillus rhamnosus* NBRC 3425	1276	99%	NR 113332.1
	IN-05	*Lactobacillus plantarum* JCM 1149	1268	99%	NR 115605.1
	IN-06	*Lactobacillus plantarum* CIP 103151	1352	99%	NR 104573.1
	IN-07	*Lactobacillus plantarum* CIP 103151	1346	99%	NR 104573.1
	IN-12	*Lactobacillus rhamnosus* NBRC 3425	1296	99%	NR 113332.1
	IN-13	*Lactobacillus rhamnosus* NBRC 3425	1301	99%	NR 113332.1
	IN-15	*Lactobacillus plantarum* CIP 103151	1353	99%	NR 104573.1
	IN-17	*Lactobacillus paracasei* NRBC 15906	1356	98%	NR 041054.1
	IN-19	*Lactobacillus plantarum* CIP 103151	1359	96%	NR 104573.1
	IN-27	*Leuconostoc mesenteroides* ATCC 8293	1263	99%	NR 074957.1

**Fig. 1.**
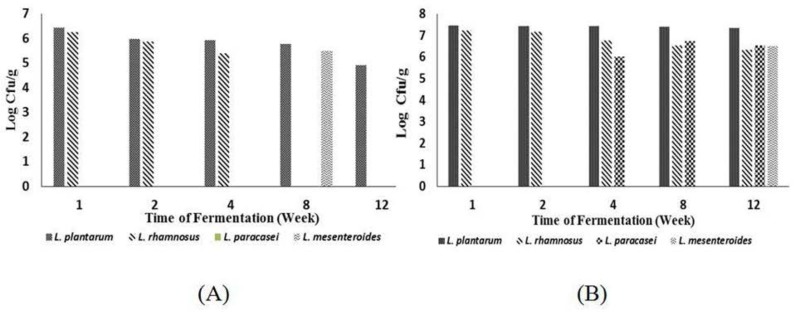
Succession of lactic acid bacteria in two types of *inasua*. *Inasua*-NS (A), *inasua*-S (B)

The bacteria underwent a succession during fermentation. In *inasua*-S fermentation, *Lactobacillus plantarum* and *L. rhamnosus* were the LAB found at the beginning of fermentation. *L. paracasei* was found after week 4 of fermentation while *Leuconostoc mesenteroides* at the end of fermentation. Dominant LAB in *inasua*-S fermentation, i.e. *L. plantarum. Lactobacillus plantarum* and *L. rhamnosus*, were also found at the beginning of *inasua*-NS fermentation. After week 8 of fermentation, *L. mesenteroides* was found. *L. mesenteroides* was also the dominant LAB at the end *inasua*-NS fermentation (Fig. 1).

**Fig. 2.**
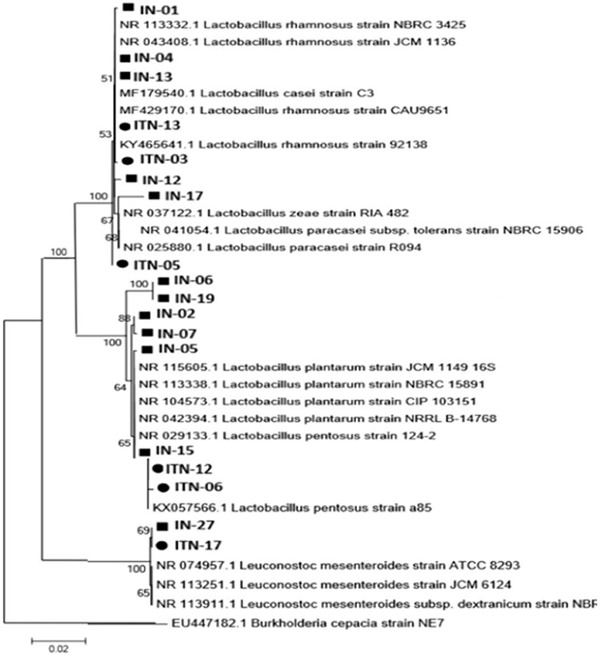
Phylogenetic tree of bacterial isolates obtained from two types of *inasua* using neighbor joining method with 1000x bootstrap replications. *Methanococcus vannielii* was as an outgroup

Phylogenetic tree analysis revealed that LAB isolates from the fermentation are related with *Lactobacillus* and *Leuconostoc*, with the exception of 3 isolates found at the end of *inasua*-NS fermentation that are closely related to *Staphylococcus* (Fig. 2).

### Metagenomic diversity.

**Fig. 3.**
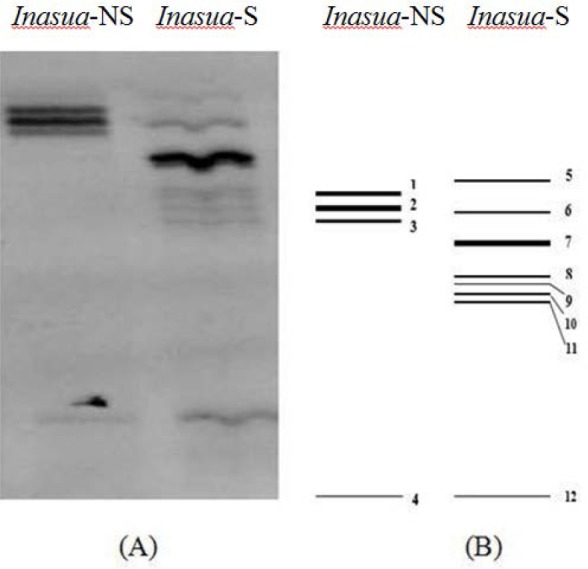
DGGE profiles of 16S rRNA from two types of *inasua* (A). Illustrations of DGGE banding patterns employing Phoretix 1D software (B)

The result of the separation of PCR products using DGGE showed that bacterial community pattern based on 16S rRNA gene varied in both samples. The pattern distribution of bacterial community from *inasua*-NS (4 bands) was less varied than from *inasua*-S (8 band) in polyacryl-amide gel (Fig. 3). Comparison with GenBank database revealed that 4 bands from *inasua*-NS were identified as *Lactobacillus curiae*, *Staphylococcus pasteuri* and two other bands were all *S. epidermidis*, while 8 bands from *inasua*-S were identified as *L. apinorum, Escherichia fergusonii, L. nagelii, L. paracasei, L. curiae* and two other bands were *L. hilgardii*. The percentage of sequence similarity between DGGE results and target genes in GenBank data was 90–99% (Table 2).

**Table 2.** Bands obtained from two types of *inasua*

**Sample**	**No. Band**	**Description**	**Length of nucleotide (bp)**	**Identity**	**Accession**
*Inasua*-NS	1	*Staphylococcus epidermidis* NBRC 100911	203	98%	NR 113957.1
	2	*Staphylococcus epidermidis* NBRC 100911	200	99%	NR 113957.1
	3	*Staphylococcus pasteuri* ATCC 51129	207	95%	NR 114435.1
	4	*Lactobacillus curieae* S1L19	202	95%	NR 109538.1
*Inasua*-S	5	*Lactobacillus sucicola* NRIC 0736	208	90%	NR 112785.1
	6	*Escherichia fergusonii* ATCC 35469	203	98%	NR 074902.1
	7	*Lactobacillus apinorum* Fhon13N	221	94%	NR 126247.1
	8	*Lactobacillus hilgardii* NBRC 15886	216	94%	NR 113817.1
	9	*Lactobacillus hilgardii* NBRC 15886	226	94%	NR 113817.1
	10	*Lactobacillus nagelii* JCM 12492	255	86%	NR 112754.1
	11	*Lactobacillus paracasei* NBRC 15889	220	94%	NR 113337.1
	12	*Lactobacillus curieae* S1L19	202	95%	NR 109538.1

**Fig. 4.**
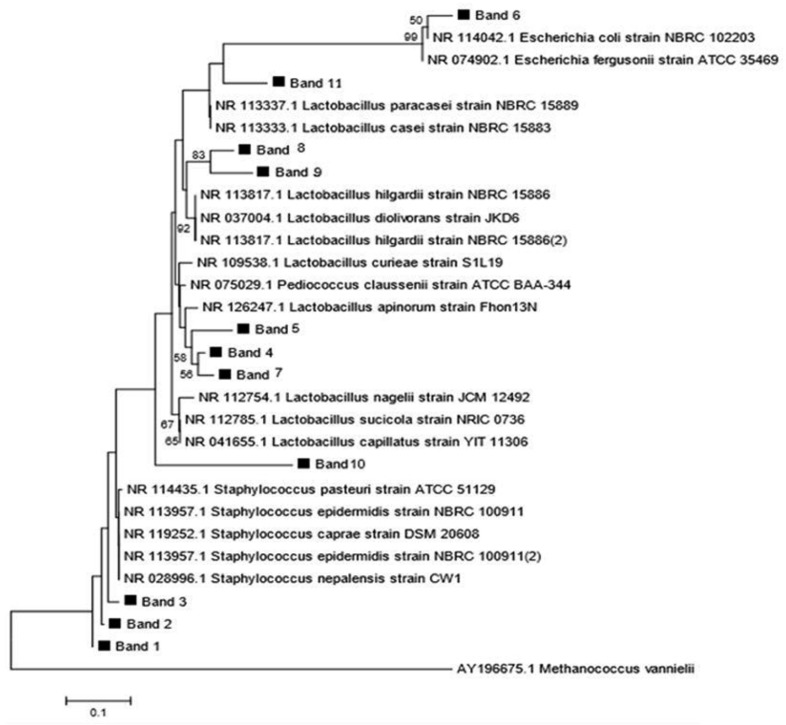
Phylogenetic tree of 11 sequences of 16S bacterial origin rRNA obtained from the DGGE analysis using neighbor joining method with 1000x bootstrap replications. *Methanococcus vannielii* was as an outgroup

Phylogenetic analysis based on 16S rRNA encoding gene was constructed using neighbor joining model with 1000× bootstrap replications and revealed that the bands found in two types of *inasua* belong to *Lactobacillus, Staphylococcus* and *Escherichia* groups (Fig. 4).

### Relative abundance and microbial diversity.

**Fig. 5.**
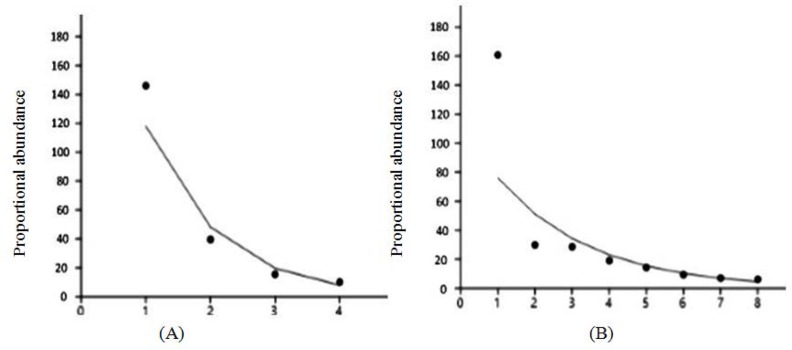
Rank of abundance curve based 16S rRNA of OTU in two types of *inasua. Inasua*-NS (A), *Inasua*-S (B)

**Fig. 6.**
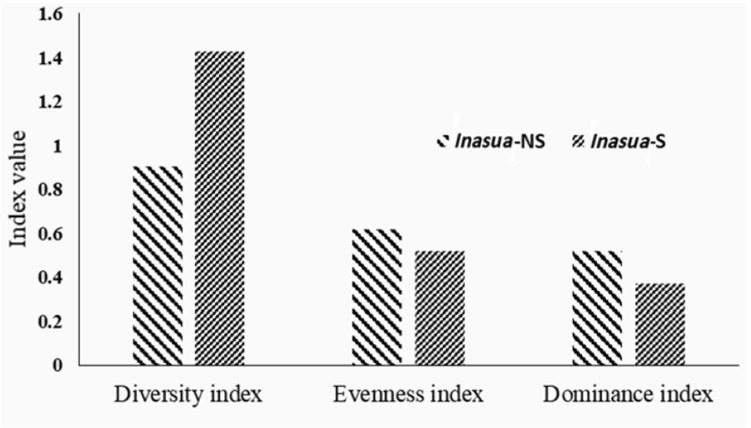
Index of diversity, evenness and dominance in two types of *inasua*

The rank of bands and abundance curve showed the relationship between richness and evenness of OTU in each *inasua* community (Fig. 5). Shannon-Wiener diversity index (H’) indicates that bacterial community diversity of *inasua*-S was 1.42 (medium), while *inasua*-NS was 0.90 (low). The evenness and dominance indices of both types of *inasua* were also different where evenness index of *inasua*-NS was 0.68 (high) and *inasua*-S was 0.52 (medium). The dominance index of *inasua*-NS was 0.52, whereas *inasua*-S 0.37 (Fig. 6).

## DISCUSSION

The succession of *Lactobacillus* and *Leuconostoc* during fermentation was strongly influenced by the characteristics of *inasua*. The factor that greatly affected the diversity of lactic acid bacteria in *inasua*-NS was salt content. In contrast, in *inasua*-S, the limiting factors that affected the microbial diversity were acid and alcohol contents (13). LAB have varying tolerance to salinity, acid and alcohol. The amount of LAB in *inasua*-NS was lower than in *inasua*-S. *Lactobacillus* is one of the LAB that can grow in high salt, acid and alcohol as well as low oxygen conditions (14). *Lactobacillus* usually grows optimally at 5–6% salt content (15) and is tolerant to pH below 4.5 (16) and 4% alcohol content (17).

*Lactobacillus plantarum* is a dominant LAB in fish (18). *L. plantarum* has the ability to use several types of amino acids as substrates, making it capable of surviving in fish with relatively low carbohydrate content (19). Its optimum growth at 4–6% salt content and pH 4–9 conditions makes it easy to find it in various ecological niches (20). *L. plantarum* plays a role in various fermented fish products from Thailand, such as *somfak* and *plasoom* that have salt content of 3–5% (21). Other LAB found in fish is *Lactobacillus rhamnosus* (18). Some of the bacterial strains have potential as probiotics (22). *L. mesenteroides* was found as dominant LAB at the end of *inasua*-NS fermentation. It is an obligate heterofermentative bacteria (23). Increasing pH at the end of *inasua* fermentation supported the growth of the LAB. *L. mesenteroides* and *L. plantarum* play roles in the production of *shikae*, a fermented fish product from Korea (24).

After week 4 of fermentation, *L. paracasei* was found in *inasua*-S fermentation. Its presence was due to the decreasing alcohol content at end of fermentation (13). *Lactobacillus paracasei* has lower tolerance to alcohol than *L. plantarum* (23). Both play a role in the fermentation of coconut sap (25). At the end of *inasua*-S fermentation, *L. plantarum* was still the dominant LAB. In addition to being a dominant LAB in fish, *L. plantarum* is also dominant in fermented sap (26).

The results of DGGE showed that most of the bacteria found in *inasua*-NS was *Staphylococcus*. Addition of more than 9% salt in fish fermentation can suppress the growth of *Lactobacillus* that plays a role in fish fermentation and supports the growth of salt tolerant pathogenic bacteria (e.g. *Staphylococcus*) (27). High salt content can inhibit the growth of spoiling bacteria, but at the same time it slows down the rate of fermentation (28). Naturally, *Staphylococcus epidermidis* and *S. pasteuri* are absent in fish. Their presence in fermented fish products is due to human contact during the preparation as well as unhygienic processing (29). Low water activity and high salt content strongly support the growth of halophilic and halotolerant bacteria, including *Staphylococcus* in fish fermentation (30). *S. epidermidis* plays a role in various fermented fish products from Thailand, such as *plara* and *nampla* where salt content of which is 11–24% (21). *L. curieae* was found in both *inasua*-S and *inasua*-NS. The LAB lives in various environments. The presence of which in several fermented products with salt indicates that the LAB can adjust in high salt condition during the fermentation.

Numerous LAB were found dominating *inasua*-S fermentation. The LAB are from fish, sap and *inasua* processing. The diversity of LAB is influenced by the sources as well as salinity, acid and alcohol contents during fermentation. The salt content below 7% can increase the growth of LAB that plays an important role in fish fermentation (27). Low pH and high ethanol contents at the beginning of fermentation were the limiting factors for the growth of pathogenic and spoilage bacteria in *inasua*-S.

*Lactobacillus apinorum, L. hilgardii, L. paracasei, L. nagelii* and *L. sucicola* are LAB found in fermented sap. The dominance of the bacteria from coconut sap in *inasua* was due to the fact that coconut sap contains simple carbohydrates easily used by bacteria rather than complex carbohydrates in fish. *Lactobacillus apinorum* is commonly found in flowers and fruits with high sugar content. The bacteria, originally isolated from the honeybee stomach, is a fructophilic LAB that tends to use fructose rather than glucose as substrate for its growth (31). Coconut sap is a source of nutrients for microbial growth because it contains high fructose (32).

*Lactobacillus hilgardii* was also found in *inasua*-S fermentation. The LAB is often found in sap fermentation because it was resistant against high alcohol contents. *L. hilgardii* produces lactic acid and grows at an optimum pH below 4.5 (33). It is a heterofermentative LAB. The ability of *L. hilgardii* to produce alcohol and various organic compounds most likely adds *inasua*-S’s sensory quality.

The other LAB found in *inasua*-S was *Lactobacillus sucicola* (*sucus* = sap). The LAB is capable of producing lactic acid through homofermentative pathways and is commonly found in fermented sap to produce traditional alcoholic beverages (34). Due to its tolerance to high alcohol and acid contents, *L. nagelii* grows in fermented sap (35). The last LAB found in *inasua*-S was *L. paracasei* that was also found in *inasua*-S fermentation with culture method since week 4 until the end of fermentation.

The uncontrolled processing of *inasua* allows the presence of microbial contaminants in fermentation. The presence of *E. fergusonii* in *inasua*-S was from non-aseptic sap tapping processor unhygienic *inasua* process. Most likely, it was from the equipment used in sap tapping (32). *E. fergusonii* can be found in low salt fish processing (36).

The OTU richness in *inasua*-NS (4 band) was lower than in *inasua*-S (8 band) due to high salt content in *inasua*-NS. The addition of coconut sap containing a number of microorganisms contributed to the high OTU richness in *inasua*-S. Dominance index of *inasua*-NS (0.52) was higher than *inasua*-S (0.37). Both types of *inasua* have one dominant OTU. *L. apinorum* and *S. epidermidis* have highest abundance of OTU in *inasua*-S and *inasua*-NS, respectively.

## CONCLUSION

The succession of lactic acid bacteria during *inasua* fermentation is strongly influenced by physicochemical characteristics of *inasua*. *Lactobacillus plantarum* was found dominant during fermentation. At the end of *inasua*-S fermentation, *L. plantarum* was found dominant, while *inasua*-NS was dominated by *Leuconostoc mesenteroides. Lactobacillus paracasei* is a LAB found only in the *inasua*-S fermentation. The result of DGGE revealed that the dominant bacteria in *inasua*-NS was *Staphylococcus*, while in *inasua*-S was *Lactobacillus*. The bacterial diversity index in *inasua*-S was higher than in *inasua*-NS.
